# Stage-specific regulation of KSHV infection by HIF-1α

**DOI:** 10.1128/jvi.00043-26

**Published:** 2026-03-10

**Authors:** See-Chi Lee, Minhchau To, Bernadett Papp, Steeve Boulant, Zsolt Toth

**Affiliations:** 1 Department of Oral Biology, College of Dentistry, University of Florida 164889, Gainesville, Florida, USA; 2 UF Genetics Institute 145773, Gainesville, Florida, USA; 3 UF Health Cancer Center 27509, Gainesville, Florida, USA; 4 UF Center for Orphaned Autoimmune Disorders, Gainesville, Florida, USA; 5 UF Informatics Institute 647919, Gainesville, Florida, USA; 6 Department of Molecular Genetics and Microbiology, College of Medicine, University of Florida 3463, Gainesville, Florida, USA; Dartmouth College Geisel School of Medicine, Hanover, New Hampshire, USA

**Keywords:** Kaposi's sarcoma-associated herpesvirus, hypoxia, HIF-1a, lytic replication, *de novo* infection, PRC2

## Abstract

The impact of physiological stress conditions on Kaposi’s sarcoma-associated herpesvirus (KSHV) infection remains poorly understood. One such stressor, hypoxia, is regulated by the transcription factor HIF-1α. We recently reported that hypoxia, or HIF-1α expression alone, can promote lytic infection in cells that typically support latent infection under normoxia. Here, we show that hypoxia-induced lytic infection is reversible, leading to an abortive lytic cycle if the hypoxic condition ceases. Additionally, we found that HIF-1α induces lytic *de novo* infection only if expressed within the first 24 h post-infection (hpi). We show that HIF-1α can bind to viral promoters and induce lytic genes only during this early window of infection, before the KSHV genome undergoes heterochromatinization and establishes latency. In contrast, regardless of the timing of HIF-1α expression during KSHV infection, the induction of HIF-1α host target genes remains unaffected. These results indicate that the heterochromatinized KSHV DNA becomes resistant to HIF-1α-mediated activation after latency is established. These findings may explain why, despite the expression of HIF-1α in Kaposi’s sarcoma tumors, KSHV remains in latency, because HIF-1α cannot induce lytic genes once the viral DNA is heterochromatinized. Importantly, we also demonstrate that inhibition of the epigenetic repressor PRC2, which associates with lytic promoters after 24 hpi, restores HIF-1α’s ability to bind viral promoters and induce lytic gene expression post-latency. Collectively, our results indicate that not only the presence of HIF-1α, but also the timing and duration of its expression during KSHV infection, are critical determinants of its ability to drive lytic infection.

**IMPORTANCE:**

The current view is that the default pathway of KSHV infection is the establishment of latency, however, how this is altered under physiological stress conditions remains largely unknown. We previously showed that hypoxia, or the expression of its transcription factor HIF-1α alone, promotes the establishment of lytic rather than latent KSHV infection. In this study, we show that the duration of hypoxia, as well as the timing and duration of HIF-1α expression, are crucial determinants in facilitating lytic *de novo* KSHV infection. Notably, we found that PRC2-mediated heterochromatin inhibits the HIF-1α-mediated upregulation of lytic genes as chromatinization of the KSHV genome progresses during infection. Our findings offer a deeper understanding of how epigenetic regulation intersects with host stress responses to influence viral pathogenesis.

## INTRODUCTION

Hypoxia is a common physiological stress condition that can modulate the outcome of viral infections (1–3). Reduced oxygen levels induce a stress response in cells that can either directly regulate viral gene expression or impact viral infections indirectly by affecting cellular metabolism or immune responses (3, 4). The major transcription factor complex governing hypoxia-induced transcriptional changes in most cells is the hypoxia-inducible factor (HIF), composed of HIF-1α and HIF-1β (5). By binding to hypoxia response elements (HREs) in promoters, HIF can recruit a wide range of transcriptional co-activators to regulate gene expression (5, 6). While HIF-1β is constitutively present in cells regardless of oxygen levels, HIF-1α is rapidly degraded through ubiquitin-mediated proteasomal degradation under normoxic conditions. In contrast, hypoxia blocks HIF-1α degradation, allowing its accumulation in the nucleus, which then induces the expression of genes that help cells to adapt to low-oxygen conditions (7).

Kaposi’s sarcoma-associated herpesvirus (KSHV) is a cancer-causing human gammaherpesvirus with a biphasic life cycle consisting of a latent and a lytic phase, both of which can be regulated by hypoxia (8, 9). We and others have demonstrated that hypoxia can regulate viral gene expression of KSHV and contribute to KSHV-associated pathogenesis, while some viral factors can, in turn, modulate the hypoxia signaling pathway (8–13). Previous reports have shown that HIF-1α binds to the promoter of lytic KSHV genes, such as the immediate-early viral gene ORF50, which encodes the lytic cycle-inducing transcription factor RTA, suggesting that hypoxia can activate the lytic phase of KSHV by inducing RTA expression (10, 13). On the other hand, it has also been reported that latent KSHV infection upregulates many hypoxia-inducible host genes, implying that KSHV may induce a hypoxic-like state in latently infected cells, thereby promoting their survival (14). The detection of HIF-1α expression in endothelial cells latently infected by KSHV in Kaposi’s sarcoma (KS) biopsies further supports the notion that HIF-1α is important not only for regulating the lytic phase, but also for maintaining latency and contributing to viral oncogenesis (9, 15, 16).

The current view is that the default pathway of KSHV infection is the establishment of viral latency, based on observations that KSHV infects most cell lines latently under ideal, stress-free cell culture conditions (17). In contrast, cells in the human body are frequently subjected to various stress stimuli, which can influence the outcome of KSHV infection *in vivo*. However, the impact of such stress signals on KSHV infection and viral pathogenesis remains poorly understood. Hypoxic conditions naturally occur in various tissues *in vivo*, such as in lymph nodes and skin, where KSHV can infect epithelial, endothelial, and immune cells (4, 18, 19). Thus, hypoxia could regulate not only latently infected cells but also the outcome of *de novo* viral infection. In this regard, we have recently demonstrated that hypoxia promotes lytic *de novo* KSHV infection of epithelial and endothelial cells, in which KSHV would otherwise establish latency under normoxia (12). In addition, we found that HIF-1α expression even under normoxic conditions can facilitate the formation of transcriptionally active chromatin at KSHV lytic promoters during infection, thereby promoting lytic infection of cells (12).

Given that, in the presence of HIF-1α expression, KSHV can exist in both a latent state (e.g., in Kaposi’s sarcoma) and establish lytic infection, this raises the question of how both infection states are possible under the same conditions (9, 12). In this study, we demonstrate that robust HIF-1α-induced lytic infection depends on the timing of HIF-1α expression during the course of KSHV infection. We also investigated whether sustained hypoxia is required for maintaining hypoxia-induced lytic *de novo* infection, and how the chromatin state of the KSHV genome influences the effect of HIF-1α on KSHV infection. Collectively, our findings provide new insights into the differential impact of HIF-1α on viral gene expression during the distinct phases of KSHV infection.

## MATERIALS AND METHODS

### Cell lines and KSHV infection

HEK293T, SLK, and iSLK (called iSLK-RTA in this study) (obtained from Jae U. Jung at the University of Southern California) cells were maintained in DMEM supplemented with 10% fetal bovine serum (FBS) and 1% penicillin-streptomycin (P/S). The 293T-BAC16 and iSLK-BAC16 cell lines were cultured in DMEM supplemented with 10% FBS, 1% P/S, and 200 µg/mL or 1 mg/mL hygromycin, respectively. BCBL-1 cells were maintained in RPMI 1640 media supplemented with 10% FBS and 1% P/S. KSHV was produced from the iSLK-BAC16 cell line (20). KSHV infection was performed as described previously (12). For KSHV infection under hypoxia, the cells were first grown in a hypoxia incubator (1% O_2_) for 16 h before KSHV was added to the cell culture medium. The infected cells were then further incubated under hypoxia. The cell culture medium was changed at 8 h post-infection (hpi), and the infected cells were harvested at the indicated time points.

### Chemical inducers and inhibitors

To induce KSHV reactivation in BCBL-1, TPA (12-O-tetradecanoylphorbol-13-acetate from Sigma) was used at 20 ng/mL concentration. KSHV reactivation in the iSLK-BAC16 cell line was induced by 1 µg/mL doxycycline (Dox from Sigma) and 1 mM sodium butyrate (NaB from Sigma). In the 293T-BAC16 cell line, KSHV lytic reactivation was induced with 3 mM NaB. EZH2 and RING1A/RING1B were inhibited using 10 μM GSK343 and 5 μM PTC209, respectively (both dissolved in DMSO), as described previously (21, 22).

### Lentivirus production and transduction

The N-terminal 3xFLAG-tagged, stabilized form of HIF-1α was expressed from pCDHCMV-MCS-EF1puro or pLVX-TetOne-Puro (Takara Bio) lentiviral vector. The stabilized form of HIF-1α contains two point mutations at Pro402 and Pro564, which prevent its degradation by VHL, resulting in its stabilization under normoxia (12). Lentivirus production and lentivirus transduction were performed as described previously (23). Two days after lentivirus transduction, the same number of cells was infected with KSHV. The expression of 3xFLAG-HIF-1α in the pLVX-TetOne-Puro lentiviral-transduced SLK cells (iSLK-3xFLAG-HIF-1α) was induced by treating the cells with 1 µg/mL Dox.

### Measurement of KSHV production

Supernatant transfer assay was used to measure the production of infectious KSHV virions. After SLK cells were grown in a hypoxia incubator (1% O_2_) or under normoxia for 16 h, they were infected with KSHV, and the supernatants were collected at 3 and 6 days post-infection (dpi). Supernatants were filtered using a 0.45 μm SFCA syringe filter, supplemented with fresh medium, and used to infect fresh SLK cells. After 24 hpi, infected cells were harvested, and total DNA was extracted for qPCR analysis to determine the level of intracellular viral DNA. Using a standard curve generated with KSHV BAC16 DNA and the measured intracellular viral DNA levels in infected cells, we calculated the number of infectious viral particles in the medium used for infection.

### Antibodies

The following primary antibodies were used in the study: anti-HIF-1α (Cell Signaling Technology 36169S), anti-ORF45 (SCBT sc-53883), anti-ORF6 (gift from Gary S. Hayward, Johns Hopkins University), anti-K8.1 (SCBT sc-65446), anti-FLAG (Sigma F1804), anti-Tubulin (Sigma T5326), anti-GAPDH (Proteintech 60004-1-Ig), anti-H3K4me3 (Active Motif 39,159), anti-H3K27me3 (Active Motif 39,155), and anti-H2AK119ub (Cell Signaling 8240S).

### Cleavage under targets and release using nuclease (CUT&RUN)

SLK cells were transduced with pLVX-TetOne-3xFLAG-HIF-1α lentivirus for 48 h, followed by KSHV infection for 72 h. The CUT&RUN assay was performed according to the manufacturer’s instructions (Cell Signaling Technology). For each reaction, 200,000 cells and 1 mg of HIF-1α antibody were used. Primers used for qPCR analysis of the CUT&RUN samples are listed in Table 1.

**TABLE 1:** List of primers used in this study

Gene	Forward (5′-3′)	Reverse (5′-3′)	Application
JMJD1A	TCTGAACGAATTGTACAGTGGCCT	AATACTCTTTGTTGATCTCCCAGA	RT-qPCR
ANKRD37	TCGCCTGTCCACTTAGCCGCAGGA	TGCTGCCTTGTGTAGTGGAGCTTC	RT-qPCR
ENO1	CTGCTCAAAGTCAACCAGATTGGCT	AGACACCATGACGCCCCAACCATT	RT-qPCR
RTApr-1.7	GATCGGCGAAGTGGATAGAGT	CCCTATTGGTCACATCTCACG	ChIP-qPCR
RTApr-1.4	TGAGGTCTATTTCCCACGACA	ACAGCTCCGACGATGAGTATG	ChIP-qPCR
RTApr-1.0	CCCCAACACAAGGACCTTTA	GCTTTTGGATACCCTGGTGA	ChIP-qPCR
RTApr-0.6	AAGACACTGACCCACCAAGG	GGTGCCACCAATGTATGACC	ChIP-qPCR
RTApr-0.1	AAAGTCAACCTTACTCCGCAAG	GCTGCCTGGACAGTATTCTCAC	ChIP-qPCR

### Total RNA and DNA extraction and their qPCR analyses

The nucleic acid extraction from cells and their qPCR analyses were performed as described in our previous publication (23). For RT-qPCR analysis of gene expression, 18S rRNA was used for normalization. Viral DNA levels were measured by qPCR using KSHV ORF11-specific primers and normalized to cellular DNA levels, which were quantified using HS1-specific primers. The new primers used in this study are listed in 5′ to 3′ orientation in Table 1, while the previously used primers are available in our earlier publication (12). RT-qPCR and qPCR results represent the average of three independent experiments. Statistical significance was assessed using a two-tailed Student’s *t*-test, with *P* < 0.05 considered significant.

### Chromatin immunoprecipitation (ChIP) assay

The ChIP assay was performed as previously described (23). For each sample, 2 μg of chromatin and 0.5 μg of antibody were used. The ChIP graphs show the average of three independent experiments. The enrichment of histone marks at specific genomic regions was calculated as the percentage of the immunoprecipitated DNA relative to input DNA. The new ChIP-qPCR primer used in this study is listed in Table 1, while the previously used primers are available in our earlier publication (12).

## RESULTS

### Sustained hypoxia is necessary for maintaining lytic *de novo* KSHV infection induced by hypoxic conditions

**Fig 1:**
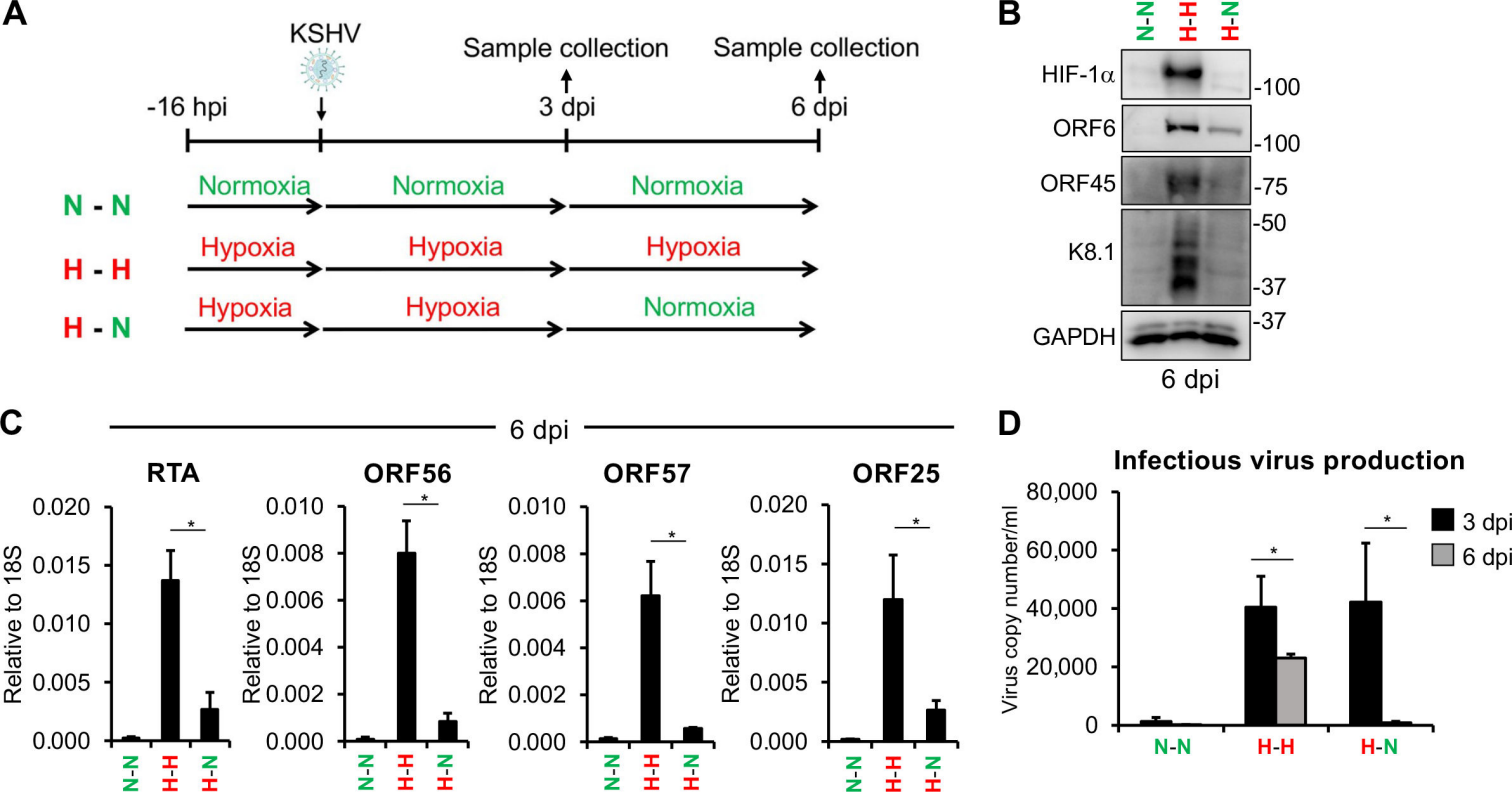
Hypoxia induces primary KSHV lytic infection, but sustained hypoxia is required to maintain the lytic cycle. (**A**) Schematic of the experimental design. (**B**) Immunoblot analysis of HIF-1α and viral protein expression at 6 dpi. (**C**) RT-qPCR analysis of viral gene expression at 6 dpi. (**D**) Measurement of infectious virus production at 3 and 6 dpi using the supernatant transfer assay. *, *P* < 0.05, sample *n* = 3.

We previously demonstrated that HIF-1α can induce the expression of RTA during *de novo* infection (12). It is also known that once RTA is expressed, it can further enhance its own expression and drive the lytic cycle of KSHV (24). This raises the question of whether sustained hypoxia is required to maintain the lytic cycle after *de novo* infection, or if the hypoxia-induced, RTA-driven lytic cycle can persist after hypoxic conditions cease. To address this question, we tested the effect of oxygen level changes on the outcome of *de novo* KSHV infection. To this end, we infected SLK cells with KSHV under hypoxia for 72 h, then changed the media and further incubated the infected cells either under hypoxia or normoxia for an additional 72 h. In addition, KSHV infection was also performed entirely under normoxia (Fig. 1A). As expected, there was no HIF-1α or lytic viral protein production in cells infected under normoxia, as HIF-1α is not stable and KSHV latently infects SLK cells under normoxia (Fig. 1B, lane 1). However, when the infection was carried out and maintained under hypoxic conditions, it led to the production of lytic viral proteins in the presence of HIF-1α at 6 dpi (Fig. 1B, lane 2), which is consistent with our previous findings (12). In contrast, HIF-1α was undetectable, and viral lytic protein expression was greatly reduced when cells infected under hypoxia for 3 days were subsequently cultured under normoxia for another 3 days (Fig. 1B, lane 3). RT-qPCR analysis also showed that cells infected under hypoxia for 6 days supported robust viral lytic gene expression, whereas the switch from hypoxia to normoxia reduced viral lytic gene expression (Fig. 1C). We also measured the infectious virus production at 3 and 6 dpi and found that, while it occurred under sustained hypoxia, switching from hypoxia back to normoxia during infection abolished virus production (Fig. 1D). These results show that hypoxia-induced lytic infection is reversible, leading to abortive lytic infection if the hypoxic stress condition is removed.

### The impact of HIF-1α on viral genes depends on the timing of its expression during KSHV infection

**Fig 2:**
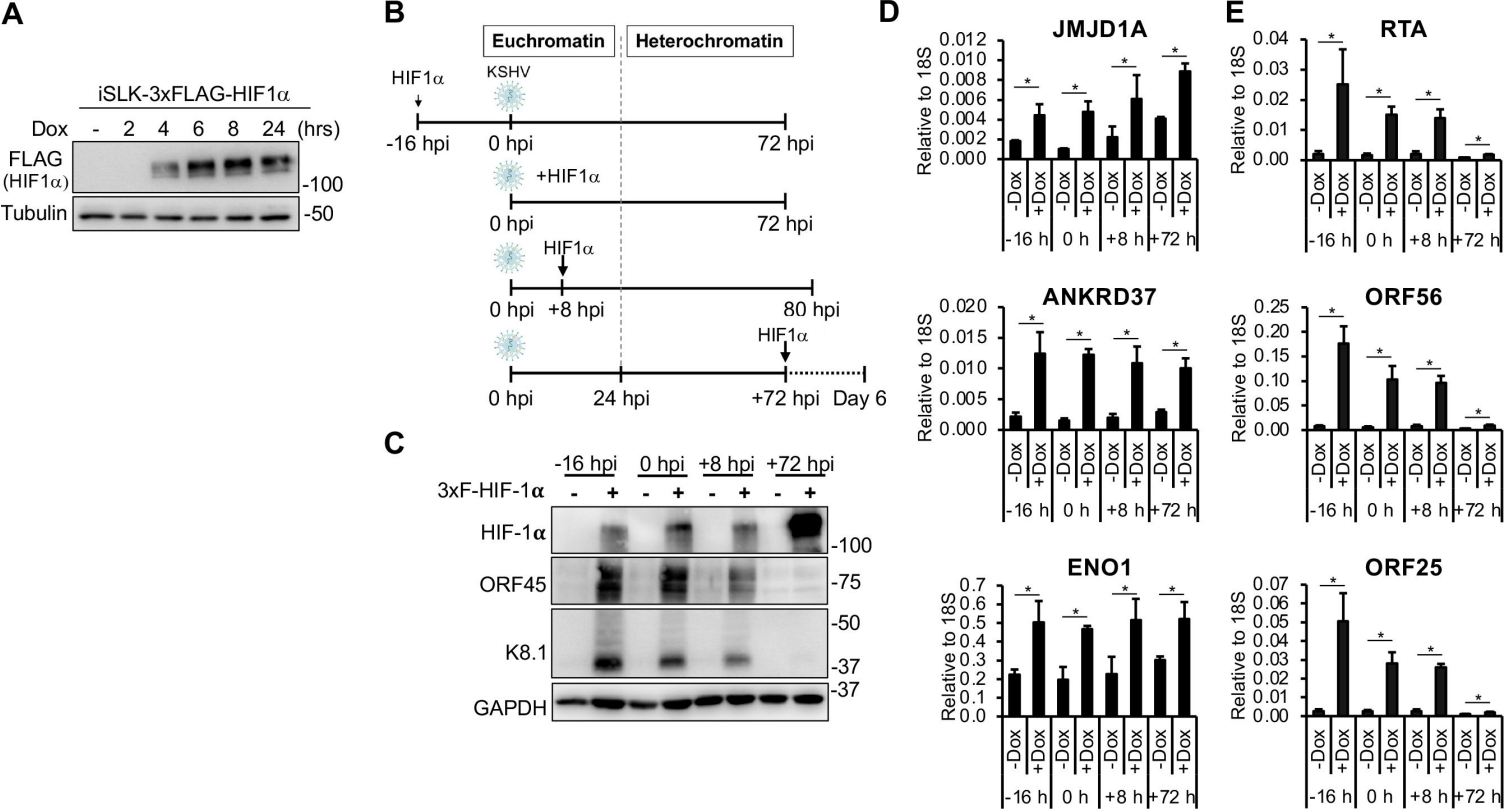
The lytic gene-inducing effect of HIF-1α depends on the timing of its expression during KSHV infection. (**A**) Immunoblot analysis of HIF-1α expression in iSLK-3xFLAG-HIF1α cells after induction with Dox for different durations. (**B**) Schematic diagram of the experimental design. 3xFLAG-HIF-1α expression was induced at different time points during KSHV infection. Cells were harvested after KSHV infection was allowed to progress for 72 h in the presence of HIF-1α expression. (**C**) Immunoblot detection of HIF-1α and viral proteins under the different experimental conditions. RT-qPCR analysis of the expression of HIF-1α target host genes (**D**) and lytic viral genes (**E**). *, *P* < 0.05, sample *n* = 3.

Given that KSHV can persist in latency or establish lytic infection in the presence of HIF-1α, we hypothesized that the ability of HIF-1α to induce lytic gene expression may depend on the timing of its expression during the course of KSHV infection. To test this idea, we generated a lentivirus expressing a Dox-inducible 3xFLAG-HIF-1α, which allowed us to express HIF-1α at specific time points during KSHV infection. We found that in lentiviral transduced SLK cells, the expression of 3xFLAG-HIF-1α could be detected as early as 4 h after Dox induction (Fig. 2A). To evaluate how the timing of HIF-1α expression influences the outcome of *de novo* KSHV infection, we performed a time-course experiment using an SLK cell line expressing Dox-inducible 3xFLAG-HIF-1α (iSLK-3xFLAG-HIF-1α). The expression of HIF-1α was induced either 16 h before infection (−16 hpi), at the time of infection (0 hpi), or at 8 or 72 hpi (Fig. 2B). Cells were harvested following 72 h of HIF-1α expression during KSHV infection (Fig. 2B). We and others previously demonstrated that during the first 24 hpi, KSHV DNA adopts a euchromatin-like structure, whereas by 72 hpi, heterochromatin forms on the viral genome and the virus establishes latency (22, 25). Thus, the expression of HIF-1α at different time points of infection also enabled us to assess whether the two chromatin states of the KSHV genome (euchromatin vs heterochromatin) during infection affect the impact of HIF-1α on lytic gene expression.

Immunoblot analysis confirmed the expression of 3xFLAG-HIF-1α in each condition (Fig. 2C). However, lytic viral proteins (ORF45 and K8.1) could only be detected when HIF-1α was expressed prior to 24 hpi (Fig. 2C). When HIF-1α was expressed at 72 hpi, a time point at which heterochromatin has been established on the viral genome and KSHV has entered latency (22), it failed to induce lytic protein expression, despite high levels of HIF-1α (Fig. 2C). RT-qPCR analysis revealed consistent induction of HIF-1α host target genes across all conditions, indicating that HIF-1α was functionally active regardless of the timing of its induction (Fig. 2D). In contrast, expression of viral lytic genes representing the immediate early (RTA), early (ORF56), and late (ORF25) classes was upregulated only when HIF-1α was expressed before 24 hpi, but not at 72 hpi (Fig. 2E). Collectively, these findings suggest that HIF-1α can promote lytic gene expression and lytic *de novo* infection only when expressed during the early stages of infection. Once latency is established and the viral genome becomes epigenetically silenced, HIF-1α appears unable to induce lytic genes.

### HIF-1α, unlike RTA, is unable to induce viral lytic gene expression after KSHV establishes latency following infection

**Fig 3:**
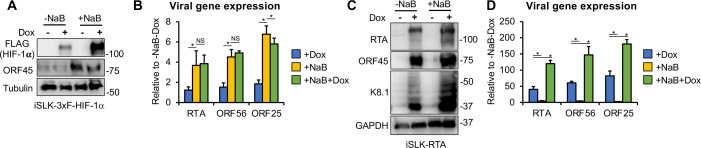
Distinct effects of the transcriptional activators HIF-1α and RTA on latent *de novo* KSHV infection. (**A**) iSLK-3xFLAG-HIF1α cells were infected with KSHV for 72 h, followed by treatment with NaB and Dox for 72 h to induce lytic reactivation and HIF-1α expression, respectively. Expression of HIF-1α and viral proteins was detected by immunoblot. Tubulin was used as a loading control. (**B**) RT-qPCR analysis of viral gene expression in the samples shown in panel (A), calculated relative to the uninduced control samples. (**C**) iSLK-RTA cells were infected with KSHV for 72 h, followed by treatment with NaB and Dox for 72 h to induce lytic reactivation and RTA expression, respectively. Immunoblot analysis of viral protein expression is shown. GAPDH served as a loading control. (**D**) RT-qPCR analysis of viral gene expression in the samples shown in panel (C), relative to uninduced cells. *, *P* < 0.05, NS: not significant, sample *n* = 3.

We compared the ability of HIF-1α and the viral transcription factor RTA to induce lytic genes after 72 hpi, the establishment of viral latency (Fig. 3). This was tested in the absence or presence of sodium butyrate (NaB), a histone deacetylase inhibitor known to epigenetically induce the KSHV lytic cycle (26, 27). We speculated that NaB might enhance HIF-1α-mediated viral gene induction, as has been shown for RTA (28). First, we infected the iSLK-3xFLAG-HIF-1α cell line with KSHV for 72 h to establish viral latency, then induced HIF-1α by treating the cells with Dox for 16 h, followed by NaB treatment for 3 days. KSHV protein and gene expression were measured 72 h after NaB treatment (Fig. 3A and B). We found that while NaB treatment induced viral lytic gene expression by four- to sixfold, HIF-1α alone did not. Interestingly, the combination of HIF-1α with NaB did not further increase lytic gene expression compared to NaB alone (Fig. 3B). In contrast, when these experiments were performed using the iSLK-RTA cell line, which expresses a Dox-inducible RTA, we found that RTA alone induced lytic gene expression by 50- to 70-fold, which further increased to 100- to 180-fold upon NaB treatment, consistent with previous studies (Fig. 3C and D) (28). Taken together, these results show that, unlike RTA, HIF-1α cannot induce viral lytic genes after viral latency has been established following infection.

### The effect of HIF-1α on KSHV lytic reactivation varies among latently infected cell models

**Fig 4:**
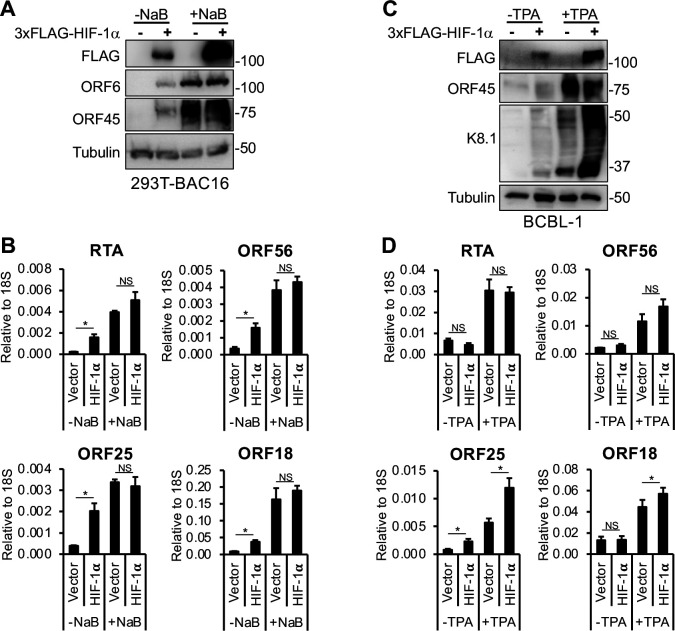
HIF-1α influences KSHV lytic reactivation differently in distinct latent cell models. (**A**) 293T-BAC16 cells were transfected with 3xFLAG-HIF-1α for 24 h, followed by treatment with 3 mM NaB for 72 h to induce KSHV lytic reactivation. Expression of HIF-1α and viral proteins was detected by immunoblot. (**B**) RT-qPCR analysis of viral gene expression in the samples shown in panel A. (**C**) BCBL-1 cells were transduced with lenti-3xFLAG-HIF-1α for 48 h, followed by treatment with 20 ng/mL TPA for 72 h to trigger KSHV lytic reactivation. Immunoblot analysis of HIF-1α and viral proteins is shown. (**D**) RT-qPCR analysis of viral gene expression in the samples shown in panel C. *, *P* < 0.05, NS: not significant, sample *n* = 3.

Previous studies have demonstrated that hypoxia can induce lytic reactivation of KSHV in latently infected cells, which requires the expression of HIF-1α (8, 29). However, whether HIF-1α alone is sufficient to trigger lytic reactivation remains unclear. To test whether HIF-1α alone can reactivate KSHV from latency under normoxic conditions, we overexpressed 3xFLAG-HIF-1α in two different long-term latently infected KSHV^+^ cell lines (Fig. 4). 293T-BAC16 is a latently infected epithelial cell line, whereas BCBL-1 is a patient-derived primary effusion lymphoma (PEL) cell line. We also assessed the potential synergistic effect of HIF-1α with two different lytic inducers (NaB and TPA) on KSHV lytic gene expression (Fig. 4).

We found that in 293T-BAC16 cells, HIF-1α induced lytic gene expression, although to a lesser extent than the lytic inducer NaB, and did not synergize with NaB to further enhance lytic gene induction (Fig. 4A and B). Interestingly, in BCBL-1 cells, HIF-1α alone induced late genes such as K8.1 and ORF25 during latency and further increased their expression in the presence of the lytic inducer TPA, while it did not enhance TPA-mediated induction of the other tested lytic genes (Fig. 4C and D). In summary, these findings show that the effect of HIF-1α on KSHV lytic reactivation and lytic gene expression varies depending on the cell type and the lytic trigger.

### During *de novo* infection, HIF-1α can bind to lytic promoters only during the early stage of KSHV infection

**Fig 5:**
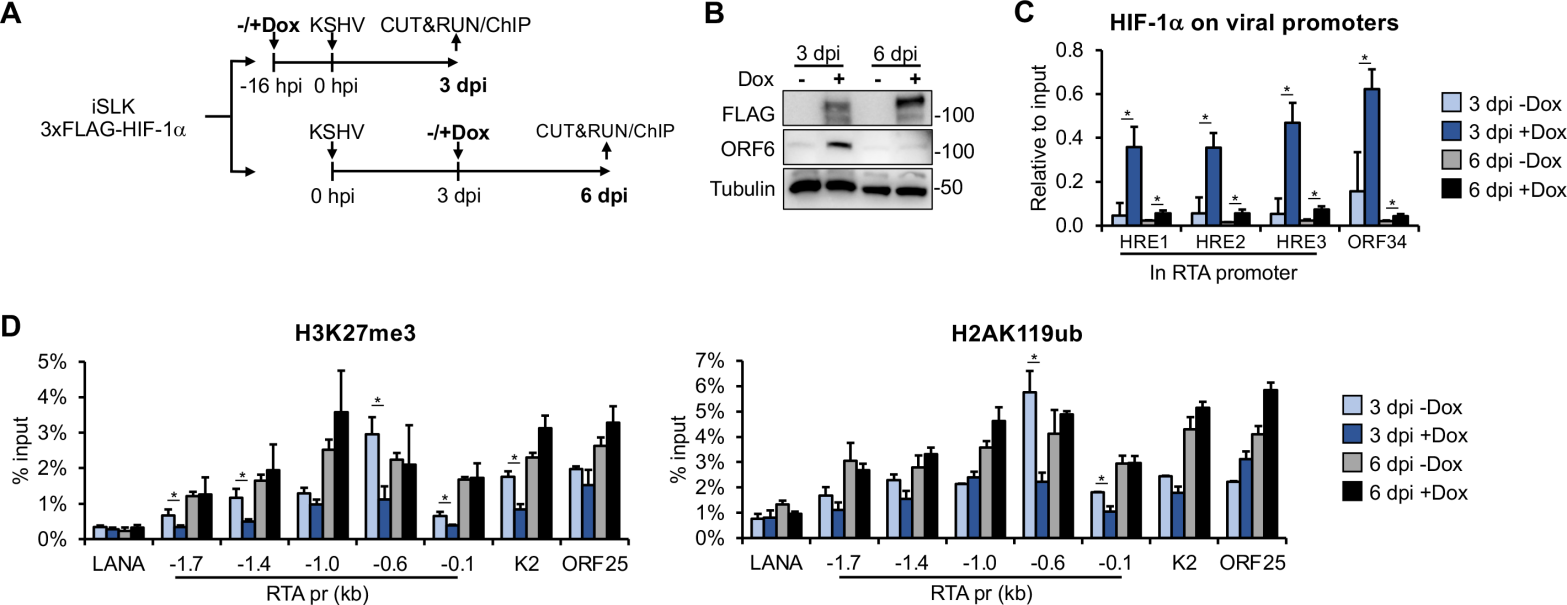
Analyzing the binding of HIF-1α to lytic promoters and its effect on viral heterochromatin during different stages of *de novo* KSHV infection. (**A**) Schematic of the experimental design. HIF-1α expression was induced with Dox either 16 h before or 72 h after KSHV infection in iSLK-3xFLAG-HIF1α cells. (**B**) Immunoblot analysis of HIF-1α and viral protein expression. (**C**) CUT&RUN analysis to detect HIF-1α binding at HREs within lytic KSHV promoters. (**D**) ChIP analysis to assess the enrichment of H3K27me3 and H2AK119ub at viral promoters. *, *P* < 0.05, sample *n* = 3.

Our data suggest that HIF-1α can promote lytic gene expression during lytic *de novo* infection, but only when it is expressed during the early stage of infection. To explain this observation, we tested the hypothesis that the incoming KSHV genome is more accessible to HIF-1α binding at lytic promoters during primary infection, whereas HIF-1α cannot bind to lytic promoters once heterochromatin has formed on the viral genome and latency is established (Fig. 5). To test this, we infected iSLK-3xFLAG-HIF-1α cells with KSHV and induced HIF-1α expression by adding Dox at either 16 h before or 72 h after infection (Fig. 5A). Following infection, the cells were cultured for an additional 72 h in the presence of HIF-1α expression. The infected cells were harvested at 3 and 6 dpi to analyze HIF-1α binding and the enrichment of heterochromatin-associated histone marks H3K27me3 and H2AK119ub at viral promoters.

Immunoblot analysis confirmed the expression of 3xFLAG-HIF-1α at both 3 and 6 dpi, while the expression of lytic viral protein ORF6 was detected only at 3 dpi, in the sample where HIF-1α was pre-expressed prior to KSHV infection (Fig. 5B). This result is consistent with our findings shown in Fig. 2. CUT&RUN assay revealed the binding of HIF-1α to three known hypoxia response elements (HREs) in the RTA promoter and one HRE in the ORF34 promoter when 3xFLAG-HIF-1α was pre-expressed before KSHV infection (3 dpi + Dox) (Fig. 5C). However, when HIF-1α was expressed 72 h after KSHV infection (6 dpi + Dox), it did not bind to the viral HREs (Fig. 5C). We also analyzed the enrichment of repressive histone marks, H3K27me3 and H2AK119ub, at the promoters of LANA, RTA, K2, and ORF25, which represent latent and lytic IE, E, and L viral genes, respectively. We found that the enrichment of H3K27me3 was reduced at lytic promoters only when HIF-1α was pre-expressed before KSHV infection (Fig. 5D). Similarly, we observed a reduction in H2AK119ub levels at the proximal promoter region of RTA only when HIF-1α was pre-expressed prior to KSHV infection. Taken together, these results support the notion that HIF-1α can bind to the promoters of lytic genes only if it is expressed during the early stage of *de novo* infection before KSHV enters latency. Once the KSHV genome becomes heterochromatinized after 24 hpi, it is no longer accessible to HIF-1α binding. The results also show that HIF-1α binding to lytic promoters is accompanied by reduced enrichment of heterochromatin-associated histone marks, which favors the expression of lytic genes, as was shown in our previous study (12).

### PRC2 inhibition facilitates HIF-1α binding to viral promoters and enhances its ability to induce lytic gene expression during latent KSHV infection

**Fig 6:**
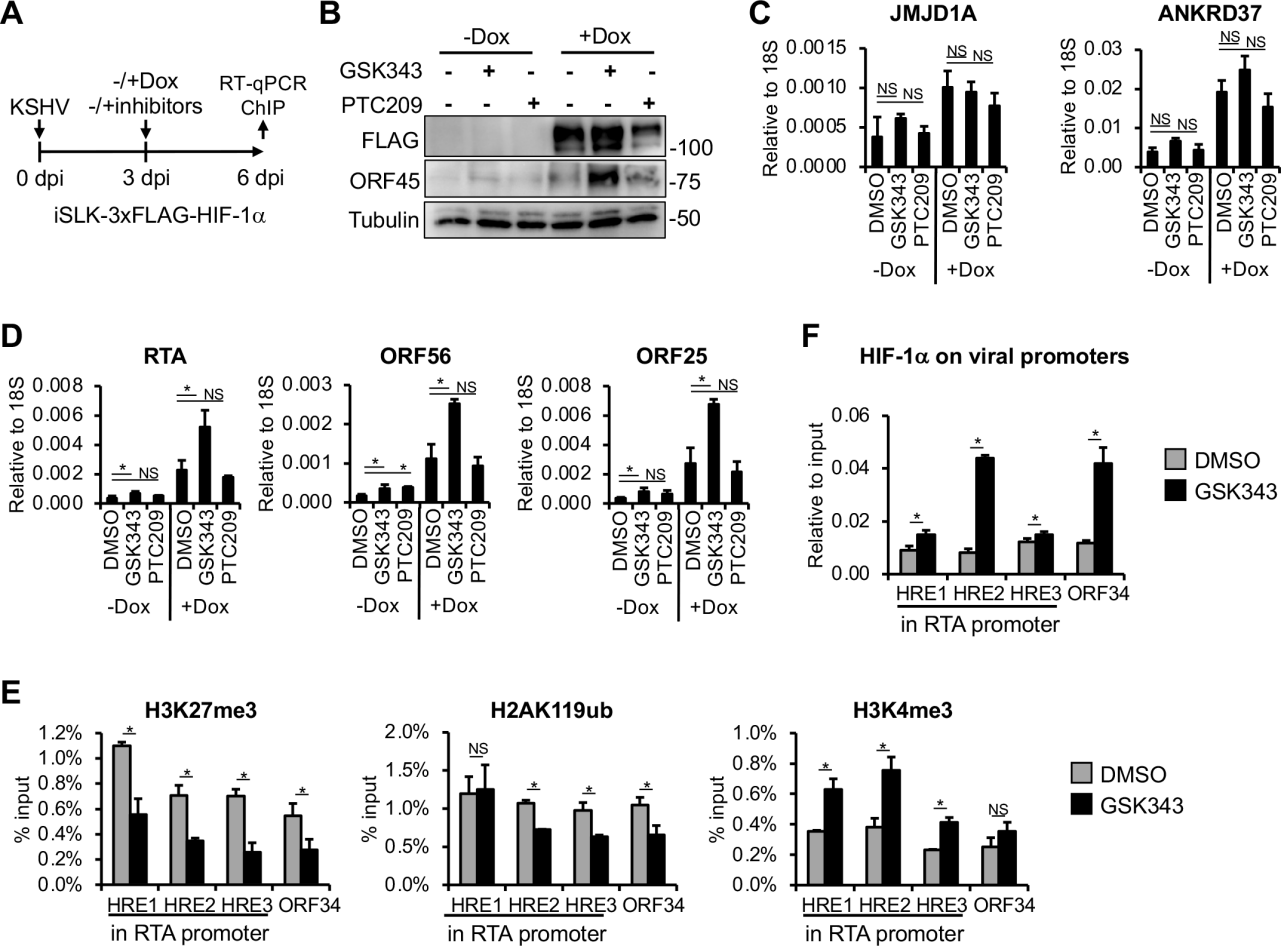
Treatment with the PRC2 inhibitor GSK343 permits HIF-1α binding to KSHV lytic promoters and induces lytic gene expression in latently infected cells. (**A**) Schematic of the experimental design. iSLK-3xFLAG-HIF1α cells were infected with KSHV for 72 h, followed by treatment with DMSO (negative control), 10 μM GSK343, or 5 μM PTC209, along with Dox to induce HIF-1α expression. (**B**) Immunoblot analysis of HIF-1α and viral protein production at 72 hpi. (**C and D**) RT-qPCR analysis of the expression of HIF-1α host target genes (**C**) and viral genes (**D**). (**E**) ChIP analysis to measure the enrichment of histone marks at KSHV lytic promoters. (**F**) CUT&RUN analysis to assess HIF-1α binding at HREs within lytic viral promoters. *, *P* < 0.05, NS: not significant, sample *n* = 3.

Polycomb repressive complexes 1 (PRC1) and 2 (PRC2) are evolutionarily conserved epigenetic repressors (30). They bind across the KSHV genome and are crucial for inhibiting lytic gene expression, thereby regulating the establishment and maintenance of latency following primary KSHV infection (22, 25, 31, 32). PRC2 and PRC1 mediate the deposition of transcriptionally repressive histone marks, H3K27me3 and H2AK119ub, respectively, which are highly enriched on KSHV lytic promoters after 24 hpi (22). We hypothesized that PRC-mediated heterochromatin on the KSHV genome might inhibit HIF-1α binding and its ability to induce lytic genes when HIF-1α is expressed after 24 hpi. To test this, we inhibited the enzymatic activity of PRC2 and PRC1 using GSK343 and PTC209, respectively, during KSHV infection, and analyzed HIF-1α binding and lytic gene expression (Fig. 6).

First, we infected iSLK-3xFLAG-HIF-1α cells with KSHV for 72 h to establish latency, then treated the cells with Dox to induce HIF-1α expression along with DMSO (negative control), GSK343, or PTC209. Seventy-two hours after Dox and inhibitor treatments, which corresponds to 6 dpi, cells were harvested for analysis of HIF-1α binding to viral promoters and viral gene expression (Fig. 6A). We found that PRC inhibitors or HIF-1α alone marginally induced viral protein expression (Fig. 6B, lanes 2–4), whereas GSK343 but not PTC209 robustly induced it when combined with HIF-1α expression (Fig. 6B, lanes 5 and 6). RT-qPCR analysis showed that neither GSK343 nor PTC209 affected the induction of cellular target genes of HIF-1α (JMJD1A and ANKRD37) (Fig. 6C). However, viral lytic gene expression was greatly increased in the presence of HIF-1α and GSK343, but not with HIF-1α and PTC209, compared to HIF-1α/DMSO control. These results suggest that PRC2 may specifically inhibit HIF-1α-induced lytic gene expression during KSHV infection (Fig. 6D). We note that GSK343 and PTC209 slightly induced lytic genes, which is consistent with previous studies showing that inhibition of either PRC2 or PRC1 upregulates lytic genes in latently infected cells (21, 22, 31, 32). Interestingly, HIF-1α alone was able to induce lytic genes to some extent, despite being expressed at 72 hpi, which seems to contradict the results shown in Fig. 2 and 3. We speculate that DMSO, which is known to affect various signaling pathways and epigenetic/transcriptional regulatory mechanisms, may have altered the viral chromatin, sensitizing it to HIF-1α-mediated induction of RTA and thereby promoting the expression of other lytic genes (33).

Given the inhibitory impact of PRC2 on HIF-1α-induced lytic gene expression, we investigated whether PRC2 affects HIF-1α binding to viral promoters. Our data show that GSK343 treatment reduced H3K27me3 levels while increasing the activating histone mark H3K4me3 at viral promoters (Fig. 6E), along with enhanced HIF-1α binding to HREs within lytic viral promoters (Fig. 6F). In summary, these findings suggest that PRC2-mediated heterochromatin at lytic promoters inhibits the binding of HIF-1α and thereby HIF-1α-mediated viral gene induction. PRC2 inhibitor treatment disrupts this repressive chromatin environment, allowing HIF-1α to bind to viral DNA and upregulate viral lytic gene expression in latently infected cells.

## DISCUSSION

**Fig 7:**
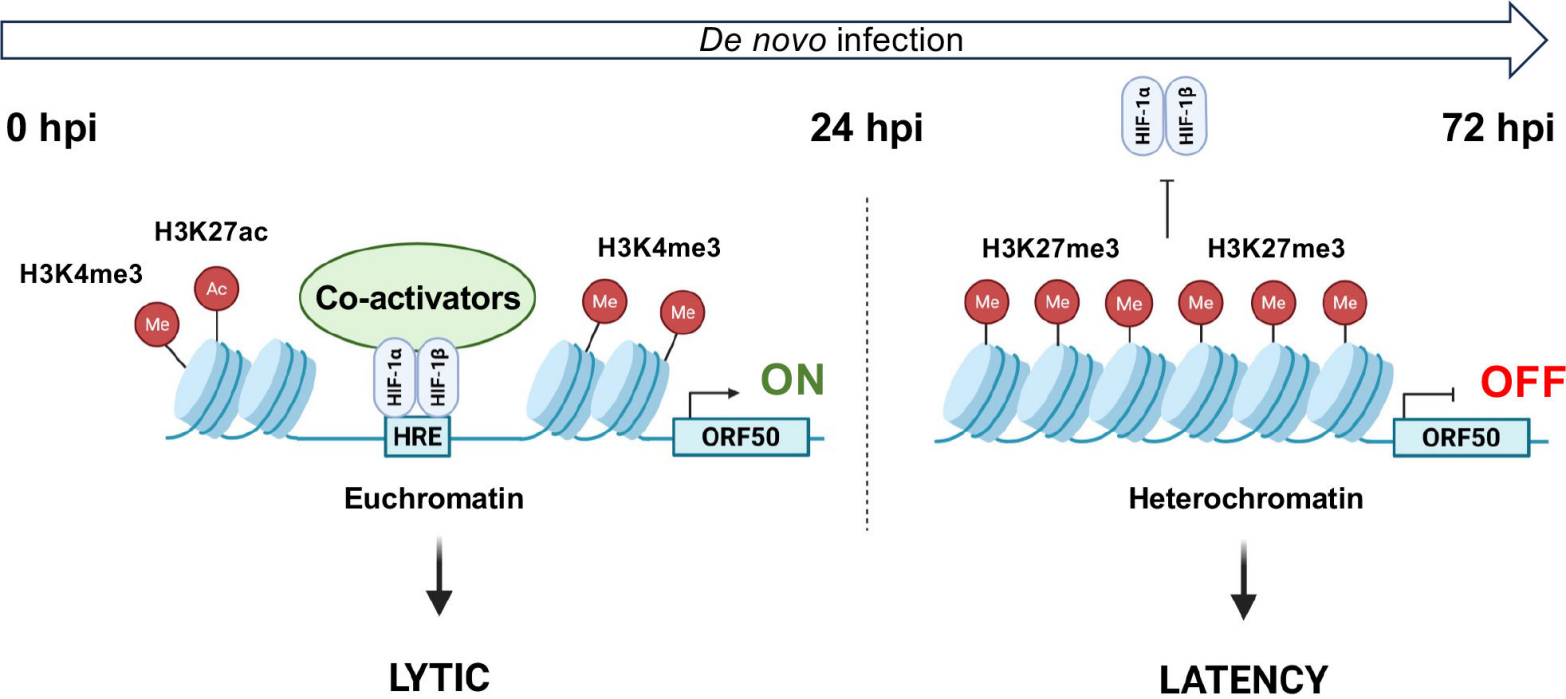
Stage-specific effect of HIF-1α on lytic gene expression during *de novo* infection. Our data suggest that HIF-1α can bind to lytic gene promoters and activate lytic gene expression (e.g., ORF50) only when it is present within the first 24 hpi, before heterochromatin is established on the viral genome. HIF-1α can recruit transcription co-activators, such as the chromatin-modifying histone acetyltransferases CBP and p300, which mediate H3K27ac and antagonize PRC2-mediated H3K27me3. However, this mechanism has not yet been confirmed in the regulation of viral promoters during KSHV *de novo* infection under hypoxic conditions. After 24 hpi, the formation of PRC2-associated heterochromatin on the KSHV genome prevents HIF-1α from accessing lytic promoters, thereby suppressing lytic gene expression and promoting viral latency, even in the presence of HIF-1α, as in the case of Kaposi’s sarcoma.

We previously showed that hypoxia, or even HIF-1α expression alone under normoxia, allows lytic *de novo* KSHV infection in cells that normally support latent infection (12). In this study, we demonstrated that the timing of HIF-1α expression during KSHV infection is critical in determining whether it can promote the lytic cycle. Specifically, we found that HIF-1α can bind to lytic gene promoters and induce lytic gene expression only when it is expressed within the first 24 hpi, prior to the establishment of heterochromatin on the viral genome and the onset of latency (Fig. 2). Our findings suggest that PRC2-associated heterochromatin formation inhibits HIF-1α binding to lytic promoters after 24 hpi (Fig. 5 and 6). Furthermore, we provide evidence that sustained hypoxic conditions are necessary to maintain hypoxia-induced lytic *de novo* KSHV infection (Fig. 1). Interruption of hypoxia during infection results in the abrogation of the lytic cycle, indicating an abortive lytic infection. This phenomenon may occur frequently in lymphoid tissues during KSHV infection *in vivo* where oxygen level fluctuations are common (34, 35). The advantage of abortive lytic infection is that the temporarily expressed lytic factors can dysregulate signaling pathways related to innate immunity, cell viability, and cell proliferation, thereby facilitating the establishment of persistent infection, while expression of lytic viral oncogenes can enhance the oncogenic potential of KSHV. Taken together with our previous work (12), our new findings suggest that not only the expression of HIF-1α, but also its timing and duration during KSHV infection, are critical for promoting HIF-1α-driven lytic infection (Fig. 7).

Previous studies have shown that HIF-1a plays a role not only in hypoxia-induced KSHV lytic reactivation in primary effusion lymphoma (PEL) cells such as BCBL-1, but also in regulating the metabolism of Kaposi’s sarcoma (KS) cells (8, 14, 29, 36). The fact that HIF-1α expression can be detected in latently infected KS cancer cells without inducing lytic cycle appears to contradict our previous finding that cells expressing HIF-1α facilitate lytic *de novo* infection (9, 12, 15). However, the current study may help resolve this apparent contradiction. We showed that after 24 hpi when KSHV DNA adopts a PRC2-associated heterochromatin (22), which is maintained during latency (31, 37), HIF-1α cannot bind to lytic promoters to induce lytic genes (Fig. 5). This suggests that heterochromatin on lytic promoters acts as a physical barrier, preventing HIF-1α from binding to DNA, as has been shown for other transcription factors in different contexts (38, 39). Supporting this idea, we observed that HIF-1α expression alone does not induce lytic reactivation in latently infected cells, unlike known lytic cycle inducing agents such as TPA or NaB (Fig. 3 and 4). This finding was consistent across three different latently infected cell lines. Among them, the KSHV-infected SLK cell line is characterized by tight viral latency, while KSHV-infected 293T cells (293T-BAC16) and BCBL-1 cells exhibit low-level spontaneous reactivation in a subset of cells (Fig. 3 and 4) (28, 40). Notably, in 293T-BAC16 and BCBL-1 cells, but not in KSHV-infected SLK cells, HIF-1α weakly induced the expression of certain lytic genes. We speculate that HIF-1α may be able to activate lytic gene expression in a subset of cells where the KSHV genome resides in a more transcriptionally permissive chromatin state, allowing HIF-1α to access its HRE target sites. In contrast, the viral transcription factor RTA can induce a full lytic cycle in latently infected cells, consistent with previous studies (Fig. 3) (41, 42). This suggests that HIF-1α, in contrast to RTA, does not function as a pioneer transcription factor capable of binding to and activating lytic promoters embedded in heterochromatin (43). In contrast, during the first 24 h of infection, the KSHV genome has a transcriptionally permissive chromatin that allows HIF-1α to bind to HREs within lytic promoters, leading to the induction of lytic genes (22, 25). Interestingly, when we examined the effect of HIF-1α on KSHV lytic reactivation in combination with different lytic triggers (NaB and TPA) across multiple cell types, we found that its impact varied depending on both the cell type and the specific inducer (Fig. 3 and 4). We speculate that the distinct mechanisms by which these lytic stimuli activate lytic genes may influence how effectively they cooperate with the HIF-1α transcriptional complex, thereby leading to variable levels of lytic gene induction. Further studies are needed to clarify how PRC2-mediated heterochromatin blocks HIF-1α binding to KSHV lytic promoters and how HIF-1α cooperates with other lytic cycle-inducing transcriptional activators to promote lytic reactivation.

Interestingly, while hypoxia can reactivate KSHV from latency in PEL cells, HIF-1α expression alone is insufficient to do so (Fig. 3 and 4) (8, 44). This suggests that, in addition to HIF-1α, other hypoxia-induced factors or hypoxia-modulated signaling pathways may also be required to trigger KSHV lytic cycle. A previous study identified one such hypoxia-induced factor, the spliced form of X-box binding protein 1 (XBP-1s), which plays a crucial role in hypoxia-induced lytic reactivation by transactivating the promoter of RTA, the viral gene that initiates the lytic cycle (44). However, whether XBP-1s cooperates with HIF-1α during hypoxia-mediated lytic reactivation or during lytic *de novo* infection remains unclear. Notably, unlike HIF-1α, overexpression of XBP-1s alone is sufficient to induce KSHV lytic reactivation in PEL cells (45). Since both HIF-1α and XBP-1s can be upregulated by various stress conditions beyond hypoxia, they may serve as important regulators of KSHV infection dynamics *in vivo* (46–49). Kaposi’s sarcoma can affect a variety of organs, including the skin, lymphoid tissues, and internal organs such as the liver, spleen, and gastrointestinal tract (50). Many of these sites are characterized by hypoxic microenvironments or are exposed to stressors that can induce HIF-1α and/or XBP-1s independently of hypoxia (e.g., cytokines, bacteria, and bacterial metabolites) (50, 51). KSHV is known to have a broad cell tropism *in vivo*, with the ability to infect epithelial cells, endothelial cells, lymphatic cells, and fibroblasts in these tissues (17). In such environments, stress-induced HIF-1α or XBP-1s may influence the course of KSHV infection, either by promoting virus dissemination or contributing to oncogenesis through the expression of lytic viral oncogenes.

Similarly to KSHV, HIF-1α has been shown to regulate both the latent and lytic phases of Epstein-Barr virus (EBV), a distinct yet closely related human gammaherpesvirus (52, 53). Hypoxic conditions promote EBV lytic activation in latently infected B cells, and hypoxia-mimetic agents that prevent HIF-1α degradation under normoxia likewise induce EBV lytic reactivation (52, 54). Despite these similarities, notable differences exist in how HIF-1α influences EBV- versus KSHV-infected cells. HIF-1α overexpression is sufficient to induce EBV lytic reactivation in latently infected B-cell lymphomas and epithelial cancer cells (52), whereas latent KSHV is comparatively refractory to HIF-1α-mediated reactivation (this study and reference 44). Mechanistically, it was proposed that HIF-1α may promote KSHV lytic reactivation by binding to the promoter of the immediate-early gene ORF50, which encodes RTA (10). In contrast, HIF-1α does not activate the EBV homolog of ORF50 but instead induces another EBV lytic switch gene, BZLF1 (52). The molecular basis for these divergent responses remains unresolved. We speculate that differences in viral chromatin architecture and in the transcription factor complexes at the KSHV ORF50 and EBV BZLF1 promoters modulate HIF-1α recruitment and activity, leading to virus-specific outcomes. However, definitive comparison of the roles of HIF factors in EBV and KSHV biology will require additional studies employing parallel analyses of both infections in the same cellular context.

The SLK cell line is an excellent model system that has yielded many new insights into the transcriptional regulation of KSHV infection. These findings have been confirmed in cell types relevant to KSHV pathogenesis, such as endothelial cells and B cells. Nevertheless, much of our current understanding of KSHV infection is largely based on studies conducted in cell cultures under ideal, stress-free conditions, which have led to the prevailing view that KSHV infection primarily results in the establishment of viral latency (17). However, this perspective may be overly simplistic, as cell culture systems do not fully replicate the environmental stress conditions *in vivo*. Consequently, there remains a significant gap in our understanding of how physiological stress stimuli regulate *de novo* KSHV infection in different physiologically relevant cell types and shape infection outcomes, such as the establishment of persistent infection and KSHV-associated oncogenesis. We propose that, in addition to HIF-1α, other transcription factors involved in stress signaling pathways may also influence the outcome of KSHV infection in a stage-specific manner, potentially depending on cell type.

## Data Availability

All data supporting the conclusions of the study are included within this article.
